# A mutation in the atrial-specific myosin light chain gene (*MYL4*) causes familial atrial fibrillation

**DOI:** 10.1038/ncomms11303

**Published:** 2016-04-12

**Authors:** Nathan Orr, Rima Arnaout, Lorne J. Gula, Danna A. Spears, Peter Leong-Sit, Qiuju Li, Wadea Tarhuni, Sven Reischauer, Vijay S. Chauhan, Matthew Borkovich, Shaheen Uppal, Arnon Adler, Shaun R. Coughlin, Didier Y. R. Stainier, Michael H. Gollob

**Affiliations:** 1 Toronto General Research Institute, University Health Network, Toronto, Ontario, Canada M5G 2C4; 2 Department of Medicine, Cardiovascular Research Institute and Division of Cardiology, University of California San Francisco, San Francisco, California 94158, USA; 3 Department of Biochemistry and Biophysics, University of California San Francisco, San Francisco, California 94158, USA; 4 Division of Cardiology, University Hospital, Western University, London, Ontario, Canada N6G 2V4; 5 Division of Cardiology, Toronto General Hospital, University Health Network, Toronto, Ontario, Canada M5G 2C4; 6 Max Planck Institute for Heart and Lung Research, Bad Nauheim 61231, Germany; 7 Peter Munk Cardiac Centre, University Health Network, University of Toronto, Toronto, Ontario, Canada M5G 2C4

## Abstract

Atrial fibrillation (AF), the most common arrhythmia, is a growing epidemic with substantial morbidity and economic burden. Mechanisms underlying vulnerability to AF remain poorly understood, which contributes to the current lack of highly effective therapies. Recognizing mechanistic subtypes of AF may guide an individualized approach to patient management. Here, we describe a family with a previously unreported syndrome characterized by early-onset AF (age <35 years), conduction disease and signs of a primary atrial myopathy. Phenotypic penetrance was complete in all mutation carriers, although complete disease expressivity appears to be age-dependent. We show that this syndrome is caused by a novel, heterozygous p.Glu11Lys mutation in the atrial-specific myosin light chain gene *MYL4*. In zebrafish, mutant *MYL4* leads to disruption of sarcomeric structure, atrial enlargement and electrical abnormalities associated with human AF. These findings describe the cause of a rare subtype of AF due to a primary, atrial-specific sarcomeric defect.

## Abstract (web summary)

Here, Michael Gollob and colleagues perform a whole exome sequencing study to identify a mutation in the atrial-specific myosin light chain gene *MYL4* in a small family with autosomal dominant familial atrial fibrillation. They also test the functionality of this *MYL4* mutation in zebrafish cardiac function and recapitulate disease-related phenotypes.

## Graphical abstract

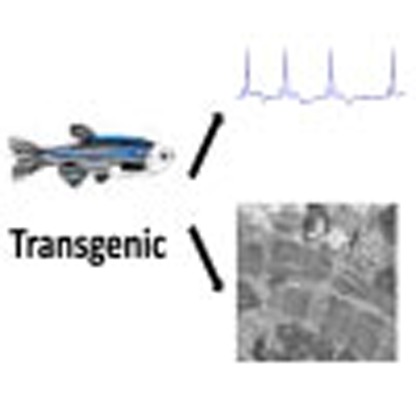

Primary cardiac electrical disorders commonly have a genetic basis and cause arrhythmias in otherwise healthy, young individuals without warning. Arrhythmias affecting the ventricle may manifest as sudden cardiac death, while atrial arrhythmias most commonly cause palpitations or stroke^1^,^2^,^3^,^4^,^5^. Collectively, genetic-based or inherited arrhythmia syndromes have become known as ‘channelopathies,' reflecting the findings that mutations in genes encoding cardiac ion channels or their chaperones are the predominant cause of these conditions to date^5^. Considerable overlap exists in ion channel genes responsible for causing arrhythmia syndromes, with genetic defects in potassium, sodium and gap junction channel genes recognized to cause episodic arrhythmias arising from either the ventricular or atrial cardiac chambers or both^6^. Typically, these channel defects alter the action potential properties or intercellular electrical communication, promoting arrhythmia vulnerability. Thus far, only a single non-ion channel gene has been identified as a cause of a primary atrial arrhythmia syndrome^7^. In 2008, Hodgson-Zingman *et al*.^7^ reported a genetic mutation in the atrial natriuretic peptide gene, *NPPA*, in a large family with atrial fibrillation (AF), and demonstrated the novel observation of the effects of this neuro-hormone on the action potential of atrial myocardium.

Herein, we describe a family with early-onset AF that remained gene-elusive for known genetic causes of familial AF. Using an *a priori* approach, we performed whole-exome sequencing in both affected and unaffected family members and identified a novel genetic variant in the atrial-specific myosin light chain gene, *MYL4*, uniquely shared only by the affected individuals. We created a zebrafish model expressing the putative mutation, and show that zebrafish harbouring the mutation exhibit a phenotype characteristic of an atrial-specific cardiomyopathy and electrical abnormalities consistent with human AF. Although genetic diseases of the cardiac sarcomere are known to lead to ventricular cardiomyopathy and associated arrhythmia^8^, a primary atrial-specific inherited myopathy presenting as an atrial arrhythmia has never previously been described.

## Results

### Study subjects

**Figure 1:**
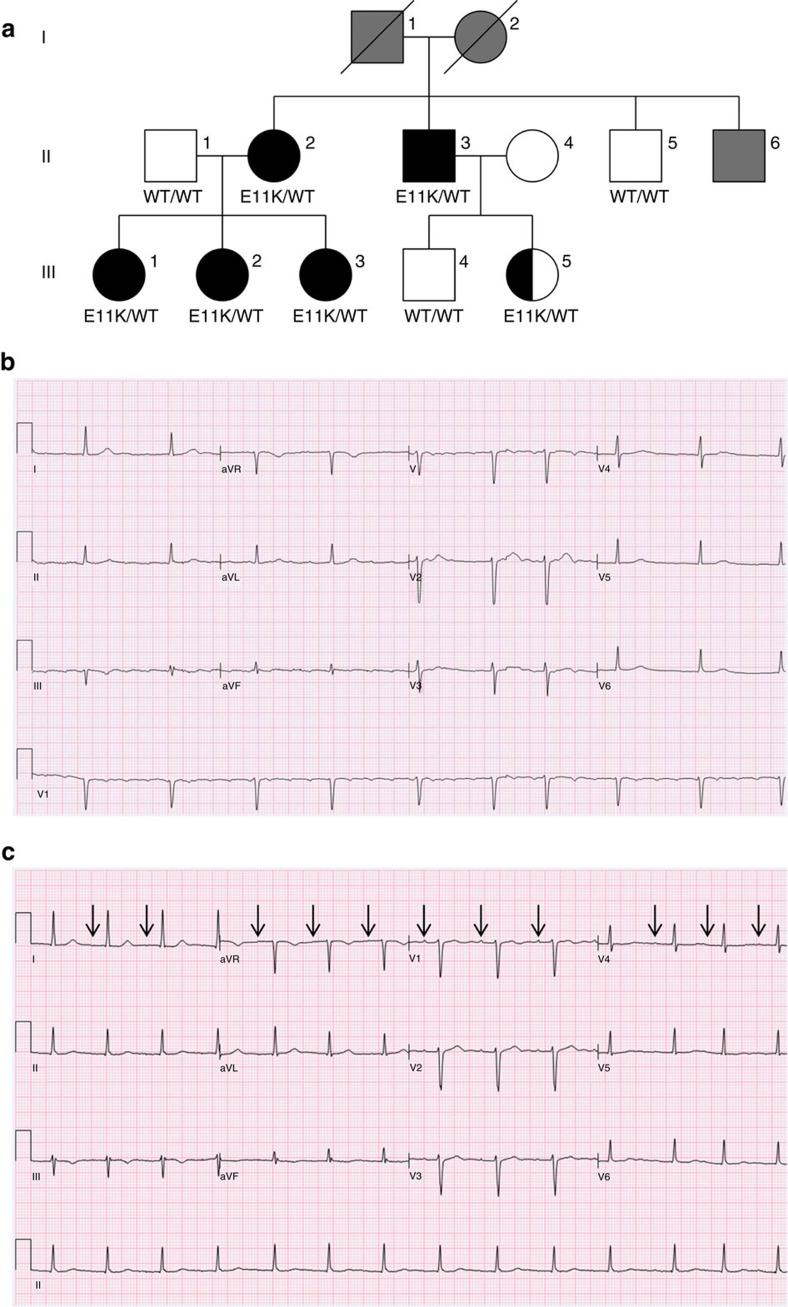
Kindred with familial atrial fibrillation caused by mutation of *MYL4*. (**a**) Kindred structure. Affected members with AF are denoted by black filled symbols; subject III.5 is half-filled, reflecting a partial phenotype. Family members with unknown phenotype are filled grey. Protein alterations encoded at the MYL4 locus are indicated. (**b**) Electrocardiogram of index case (subject III.3) demonstrating atrial fibrillation with slow ventricular rate response while off medication. (**c**) Electrocardiogram of sister of the index case (subject III.2) during normal sinus rhythm. Arrows point to very low amplitude P-waves. Prolonged PR interval (atrial–ventricular conduction time) is also present.

The index case in our study (Fig. 1a, III.3) presented with palpitations at 26 years of age, and revealed a family history of AF. Electrocardiography (ECG) showed AF with slow ventricular rate response (Fig. 1b). During periods of sinus rhythm and before medical therapy, extremely low amplitude P-waves and prolonged atrial–ventricular conduction (first-degree AV block) were observed, consistent with impaired atrial conduction. The patient had frequent recurrences of AF despite medical therapy. An intra-cardiac electrophysiological study demonstrated large areas of electrical silence within the left atrium, and some regions of the left and right atria showed rapid electrical signals consistent with ongoing atrial arrhythmia. A catheter ablation was performed. Despite a successful procedural end point, the patient continued to experience paroxysmal AF.

Family history revealed two additional symptomatic relatives. The patient's mother (Fig. 1a, II.2) had first presented with AF at the age of 32 years and had a permanent pacemaker placed at the age of 48 years for bradycardia in the setting of permanent AF. Echocardiography revealed an enlarged left atrial volume index (LAVI; 40 ml m^−2^; normal <28 ml m^−2^), a profoundly decreased left atrial function index (LAFI)^9^ and normal biventricular size and function (Table 1). A 25-year-old sister was then investigated for complaints of palpitations (Fig. 1a, III.2). Rhythm monitoring confirmed frequent episodes of AF. During sinus rhythm, consistent with the observations of her sister, electrocardiography demonstrated very low amplitude P-waves and first-degree AV block (Fig. 1c). In addition, 48-h ambulatory heart rate monitoring showed profound sinus bradycardia with sinus rates as low as 33 beats per minute (b.p.m.; Table 1). Echocardiography showed an enlarged left atrium (LAVI 38 ml m^−2^) and decreased LAFI. Arrhythmia screening was offered to asymptomatic relatives, including another sister, two maternal uncles and two first cousins. Interestingly, a 22-year-old sister (Fig. 1a, III.1) and a 50-year-old uncle (Fig. 1a, II.3) were found with permanent AF, and a 20-year-old cousin (Fig. 1a, III.5) had an electrocardiogram also exhibiting low amplitude P-waves and first-degree AV block as observed in the index case, but no AF to date (Table 1). Overall, the predominant clinical phenotype represented in the family consists of AF, conduction disease and reduced left atrial function as measured by LAFI. All mutation carriers showed clinical penetrance, although full disease expressivity appears to be age-dependent, as subject III.5 at the age of 20 years has not yet shown evidence of AF or decreased left atrial function. Clinical details of the family are shown in Table 1.

### Genetic analysis

Following genetic testing of the family which confirmed the absence of mutations in genes known to cause familial AF (*GJA5*, *KCNA5*, *KCNE2*, *KCNJ2*, *KCNQ1*, *KCNH2*, *NPPA* and *SCN5A*), the possibility of a mutation in a novel gene was considered. Whole-exome sequencing was performed in parallel on five clinically affected individuals (subjects II.2, II.3, III.1, III.2 and III.3), two unaffected family members (II.1 and II.5) and 10 arrhythmia-free, healthy control subjects. Bioinformatic analysis was aimed to identify novel protein-altering genetic variants unique to the affected individuals and absent from unaffected family members, parallel controls and large public exome databases. Six novel protein-altering variants were identified to be uniquely shared among the five affected individuals, including three variants that are highly conserved among species (Supplementary Table 1). Of these variants, only one, *MYL4* p.Glu11Lys (E11K), affected a gene highly expressed in cardiac tissue—specifically, in atrial myocardium (Supplementary Table 2). *MYL4* encodes the atrial-specific myosin essential light chain (ELC), a key sarcomeric component^10^. The E11K mutation resides within the protein's N-terminus, a region demonstrated to bind F-actin^10^,^11^. Additional comprehensive sequencing of the coding region of *MYL4* from DNA of 100 unrelated individuals with AF and no other cardiac co-morbidities did not yield any rare variants in *MYL4*.

### Zebrafish modelling

To test pathogenicity of this *MYL4* mutation, we generated a transgenic zebrafish line expressing the corresponding mutation in the zebrafish ortholog *cmlc1* under control of the atrial-specific *myh6* promoter (E17K transgenic). While the zebrafish heart is two-chambered, heart rate, P-wave duration, and PR, QRS and QT intervals are similar to those of humans^12^,^13^. A separate transgenic line expressing wild-type (WT) *cmlc1* served as a control (WT transgenic). Quantitative PCR showed no statistical difference in *cmlc1* expression between E17K transgenics and WT transgenics at larval stages, with decreased expression in the E17K adult (Supplementary Fig. 1). In addition, we assayed the gene expression profile of 13 other genes specific to atrial myocardial function, including major subunits of the L-type calcium channel (LTCC; *cacna1c* and *cacna1d*), the cardiac ryanodine receptor (*ryr2b*) and sarcomeric proteins (*tnnc1a*, *tnnc1b*, *myh6* and *actn2b*). No difference in gene expression was observed for the selected calcium-related genes. A modest but statistically significant increase in expression was shown for troponin C genes *tnnc1a* and *tnnc1b* in the E17K transgenic adult atrium versus WT transgenic (*P*=0.003 and *P*=0.001, respectively; Supplementary Fig. 1). Whether this upregulation in troponin C in the E17K mutant represents a compensatory secondary change is uncertain.

**Figure 2:**
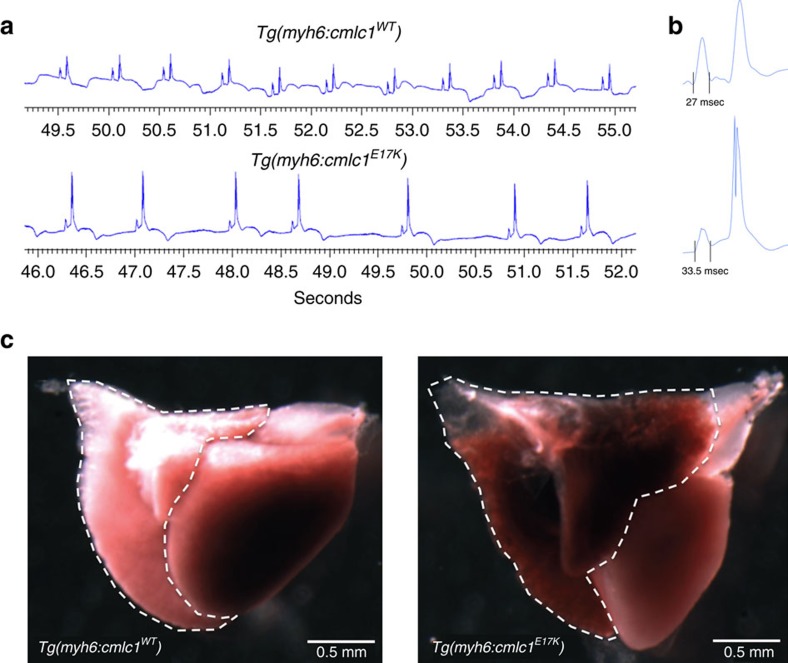
Electrocardiograms and whole hearts from WT transgenic and E17K transgenic zebrafish. (**a**) WT transgenics exhibit a regular heart rate with electrocardiogram parameters typical for adult zebrafish. E17K transgenics display significantly slower sino-atrial activity and increased heart rate irregularity compared with controls. (**b**) Signal-averaged P-wave duration is prolonged in E17K transgenics, indicative of slower atrial conduction time. (**c**) Atrial area has been traced in isolated whole-WT transgenic and -E17K transgenic hearts (dotted lines), showing enlarged atrial chamber size in E17K transgenic.

Electrocardiography was performed on control and experimental adult fish. E17K transgenics exhibited specific electrocardiographic abnormalities recognized as surrogate risks for human AF (Table 2)^14^,^15^. Abnormalities included irregular sino-atrial impulse generation and bradycardia, and prolonged P-wave duration, reflecting slowed atrial conduction (Fig. 2a,b). Eight of 11 (73%) of E17K transgenics exhibited severe bradycardia (in fish, HR<80 b.p.m.) compared with 0 of 10 controls. E17K transgenics exhibited a 25% decrease in heart rate compared with controls (*P*=0.004, Mann-Whitney *U* test).

We evaluated whether E17K transgenic fish exhibited atrial enlargement consistent with atrial myopathy. Hearts were dissected from freshly euthanized adult fish and atrial and ventricular surface areas were measured. No statistical difference in ventricular surface area was observed between control and experimental fish (1.3±0.3 mm^2^ versus 1.6±0.2 mm^2^). In contrast, atrial areas were increased 1.6-fold for experimental fish compared with controls (1.74 versus 1.03 mm^2^, *P*=0.007; Fig. 2c). Strikingly, E17K transgenic atrial size exceeded ventricular size (atrial:ventricular area ratio 1.41 versus 0.65 for controls, *P*=0.01).

**Figure 3:**
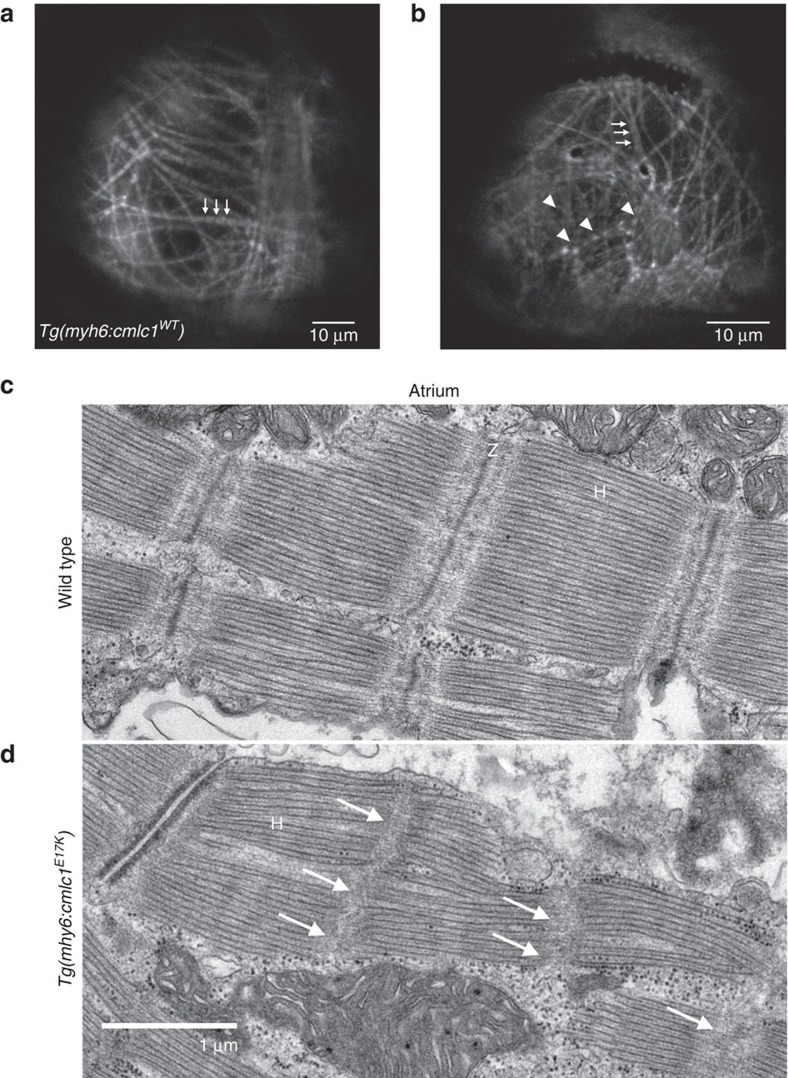
Myofibrillar organization and sarcomeric structure in WT and E17K transgenics. (**a**) A WT transgenic atrium at larval stage (5 d.p.f.) in the *Tg(myl7:LifeAct-GFP)*^*s974*^ background shows GFP-labelled F-actin, with myofibrils organized linearly across the atrium, either up-and-down or left-to-right. The myofibrils have a ‘dotted line' pattern, with well-demarcated box-like shapes indicating normal arrangement of F-actin in the myofibrils. The non-fluorescent gaps (arrows) represent the actin-poor H-zones. (**b**) In an E17K transgenic larval atrium, some myofibrils appear unaffected (arrows), but there are large areas where myofibrils are not linearly organized and actin localization appears abnormal, giving a stippled appearance to the sarcomeres without clear H-zones (arrowheads). (**c**) Electron microscopy of adult WT zebrafish atrium shows clear H-zones and Z-disks. (**d**) Electron microscopy of E17K transgenic atrium shows preserved H-zones but absent Z-disks (arrows). H, H-zone. Z, Z-disk.

We then investigated whether the observed heart rhythm and atrial enlargement in E17K transgenic fish were correlated with sarcomeric disruption in larval or adult fish, given Myl4's presence in atrial sarcomeres. WT transgenic and E17K transgenic fish were crossed into the *Tg(myl7:LifeAct-GFP)*^*s974*^ transgenic line, which labels myocardial F-actin with GFP and allows live imaging of myofibril organization^16^. In WT transgenic atria, imaged at 5 days post fertilization (d.p.f.), normal myofibril organization was seen (Fig. 3a). Compared with controls, myofibrils in E17K transgenic atria appeared disorganized, and in some myofibrils F-actin corresponding to individual sarcomeres appeared stippled and blurred, indicating abnormal organization of H-zones and Z-disks (Fig. 3b). Electron microscopy on adult zebrafish hearts was also performed, using WT hearts as a control. In contrast to WT, E17K transgenic atria had largely absent Z-disks (Fig. 3c,d).

Finally, we evaluated whether zebrafish myocardial function was affected before adulthood. Heart rate, and atrial and ventricular fractional shortening were measured for E17K and WT transgenic larvae at 3, 5 and 10 d.p.f. (Supplementary Fig. 2). There were no statistically significant functional differences between groups, suggesting that myofibrillar disarray, as described above, precedes atrial dysfunction and arrhythmia.

### Structural modelling

Myl4 and other myosin ELCs from vertebrate striated muscle have an additional ∼40 N-terminal amino acids that is absent in smooth muscle isoforms^11^,^12^. Although the crystal structure for shorter ELCs has been solved^11^, the structure of the extended N-terminus, where our identified mutation resides, is unsolved. Chemical cross-linking and deletion mutagenesis studies of the extended N-terminus have identified a major actin-binding site involving the first 11 residues of Myl4 (refs 11, 17, 18). This highly charged N-terminus binds directly to a cluster of C-terminal acidic residues at positions 360–364 of F-actin^19^,^20^,^21^. The interaction between the Myl4 N-terminus and actin is considered to be electrostatic, since increasing the ionic strength abolishes or significantly reduces the amount of crosslinking^17^,^21^,^22^.

**Figure 4:**
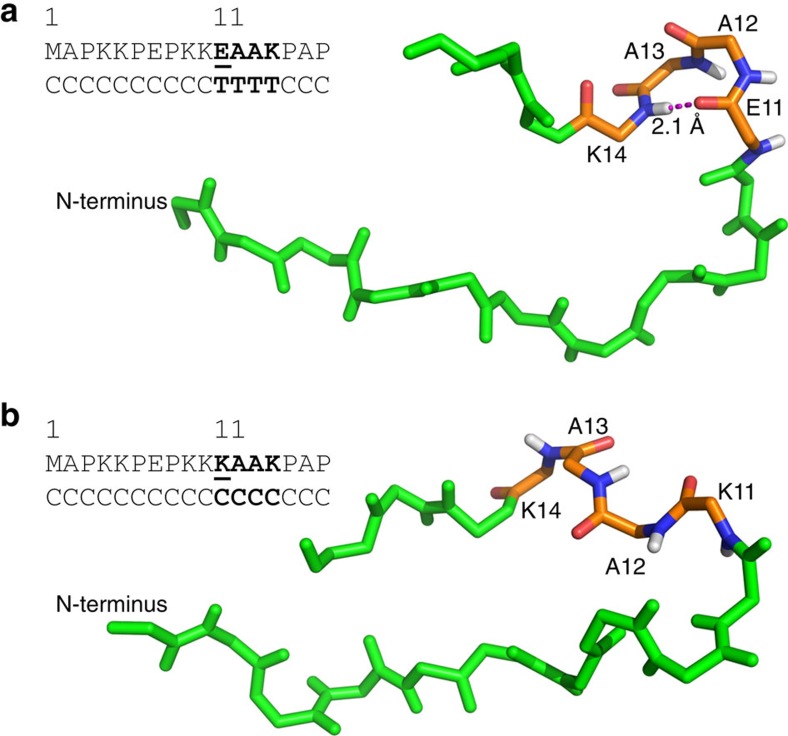
Secondary structure prediction for Myl4 WT and mutant N-termini. (**a**) In the presence of WT E11, a type II β-turn is predicted with the formation of a stable hydrogen bond between E11 and K14. (**b**) Substitution of positively charged K11 for E11 results in loss of the predicted β-turn. C, coil; T, β-turn.

**Figure 5:**
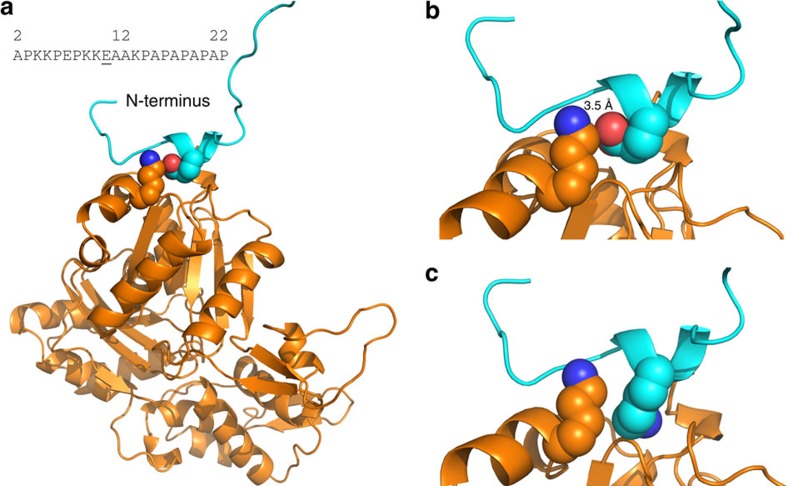
Predicted interaction of Myl4 with putative actin-binding site. (**a**) WT E11–Myl4 is in close proximity to K358 of actin and is predicted to form a stable electrostatic interaction between the negatively charged oxygen of E11 and positively charged nitrogen of actin K358 (**b**). (**c**) This electrostatic interaction within the actin-binding site is lost with substitution of positively charged K11. Dark-blue sphere, positively charged nitrogen molecule. Red sphere, negatively charged oxygen molecule.

A secondary structure model^23^ of the Myl4 N-terminus predicts a β-turn at residues 11–14, stabilized by hydrogen bond formation between residues E11 and K14 (Fig. 4a). Substitution of negatively charged glutamate (E11) for positively charged lysine (K11) predicts loss of the β-turn secondary structure (Fig. 4b). We next modelled the predicted quaternary structure and electrostatic interaction between E11–Myl4 and F-actin. An electrostatic interaction within a 5 Å distance is predicted to occur between negatively charged E11–Myl4 and K358 of actin—an interaction that is lost with the substitution of K11 in Myl4 (Fig. 5a,b). Thus, alterations in secondary structure and electrostatic interactions caused by E11K may impair direct binding of Myl4 to actin, thereby altering the sarcomere structure and function.

## Discussion

The phenotypes seen in human and zebrafish studies highlight the sarcomere as a centre for mechano-electrical integration. E17K transgenic zebrafish exhibited myofibrillar disarray, and electron microscopy showed absent Z-disks. Importantly, Z-disks couple electrical excitation to contraction with direct communication between the sarcolemma and the sarcomere via T-tubules formed through cell membrane invaginations^24^,^25^. T-tubules have a high density of LTCCs, and immunofluorescence confirms localization of LTCCs directly to Z-disks^25^,^26^. As the major mediator of calcium influx during phase 2 of the cardiac action potential, LTCC activation triggers ryanodine receptor activation leading to further release of calcium from the sarcoplasmic reticulum, and subsequent sarcomere activation and contractility^26^,^27^. Finally, F-actin is a core component of Z-disks. F-actin crosslinks adjacent sarcomeres via binding with α-actinin, allowing for uniform transmission of force across sarcomeres. Taken together, our findings suggest that the identified E11K-*MYL4* mutation leads to destabilization of the F-actin–Z-disk complex, which may impair calcium signalling and cause atrial myopathy, leading to atrial arrhythmias. Given our findings, it is possible that more cases of early-onset AF may have features of subclinical atrial myopathy than previously recognized.

This study describes a previously unreported autosomal dominant form of heritable AF, with early age of onset, associated conduction system disease and atrial myopathy. It is compelling that a recent study describing large-scale whole-genome sequencing of the Icelandic population has identified a founder frameshift mutation in *MYL4* present in 1.5% of the Southern Icelandic population that is associated with AF as a homozygous genotype^28^. Although we cannot exclude a potential contributing role to phenotype of the five other identified novel variants shared among affected individuals, their effect on an atrial-specific phenotype would be expected to be minimal at most, in light of their highest gene expression in tissues outside of the heart and absence of any currently known role in myocardial electrical or contractile function (Gene Ontology, Supplementary Table 2). Altogether, these findings and the modelling of E11K*-MYL4* in zebrafish strongly support a causal relationship of *MYL4* mutations for a unique subtype of human AF.

## Methods

### Research subjects

All patients provided written informed consent for genetic analysis and clinical records review under an approved research protocol from the University of Ottawa Heart Institute Research Ethics Board. ECG measurements, left ventricular ejection fraction and LAVI using biplane method of discs were performed according to the accepted clinical guidelines. LAFI was measured as described^29^.

### Genetic analysis

DNA was prepared from venous blood or saliva samples from the index case and kindred members. Exome sequencing of five clinically affected and two unaffected kindred members, as well as 10 unaffected, unrelated controls was performed on the Agilent SureSelect 51 Mb exome capture kit, followed by 100-bp paired-end sequencing on the Illumina HiSeq 2000 platform to achieve high coverage. Sequencing generated a mean coverage of 64-fold, with 79 and 87% of target sequences at >20 × and 10 ×, respectively. Sequences were aligned to Human Genome version 19 (NCBI 37) using the Burrows–Wheeler Aligner. Single-nucleotide variant and indel calls were assigned quality scores using Genome Analysis Toolkit version 2.2 and annotated for novelty (using dbSNP, 1000 Genomes Project and NHLBI exome databases). Bioinformatic analysis was aimed to identify rare (MAF<0.01%) or novel protein-altering genetic variants unique to the affected individuals and absent from unaffected family members, parallel controls and large public exome databases. Novel, protein-altering variants uniquely shared among the five clinically affected individuals were all confirmed through PCR amplification and Sanger's DNA sequencing. Conservation scores of novel variants were assigned a maximum score of 1 using PhastCons^29^ to quantify conservation in reference to 33 mammalian taxa. Mutation Taster^30^ and Polyphen-2 (ref. 31) softwares were used to predict the effect of protein-altering variants on protein structure and function. Gene ontology and tissue expression for each variant was summarized using the Gene Ontology Consortium (www.geneontology.org) and GeneCards (www.genecards.org) databases, respectively.

### Transgenic zebrafish creation

The zebrafish *MYL4* orthologue, *cmlc1*, was used as a template to create the corresponding E11K mutation using site-directed mutagenesis. WT and mutant *cmlc1*-coding sequence was cloned into an I-SceI vector under the control of the atrial myosin heavy chain (*myh6*) promoter; the vector also contained mCherry under the control of a crystallin alpha A (*cryaa*) promoter. Transgenesis was performed in TL background as described^32^. *Tg(myh6:cmlc1*^*E17K*^*,cryaa:mcherry)*^*s977*^ (termed E17K transgenic) and *Tg(myh6:cmlc1*^*WT*^*,cryaa:mcherry)*^*s978*^ (termed WT transgenic) zebrafish were maintained under standard conditions.

### Zebrafish ECGs

Electrocardiograms were performed for 2 min on anaesthetized adult transgenic zebrafish as previously described^33^, except that the two electrodes were placed under the left pectoral fin and on the pericardium, respectively. An Iso-DAM8A amplifier (World Precision Instruments Inc., Sarasota, FL) and Power 1401 data-acquisition interface (Cambridge Electronic Design, Cambridge, England) were used to acquire data. Data were analysed using Spike2 software, version 5.19 (Cambridge Electronic Design) and LabChart 8 (ADInstruments, Sydney, Australia). Fish that exhibited an injury current (ST elevation) on ECG were considered to have poor placement of electrodes and were excluded from analysis. For each fish, 10 consecutive heartbeats where a clearly identifiable baseline was seen were used to determine the average RR interval, PR interval and P-wave duration. Ten WT transgenic and 11 E17K transgenic fish were studied. Heart rate variability was calculated from the standard deviation of RR intervals.

### Zebrafish adult heart measurements

For atrial and ventricular area measurements, adult zebrafish hearts were recovered as previously described^16^. Atrial and ventricular surface areas were measured using Fiji software (www.fiji.sc/). Fifteen WT transgenic and eight E17K transgenic fish were analysed. An unpaired two-tailed *t*-test was used to determine statistical significance between groups. *P* values of≤0.05 were considered statistically significant.

### Zebrafish heart larval imaging and functional measurements

For live larval zebrafish myofibril imaging, WT transgenic and E17K transgenic fish were crossed into the *Tg(myl7:LifeAct-GFP)*^*s974*^ background to allow for imaging of myofibril organization as previously described^16^. A total of seven WT transgenic and eight E17K transgenic zebrafish were imaged. All experimental results were analysed in a blinded fashion.

Zebrafish larvae have hatched and swim independently. Larval heart rate and fractional shortening was performed by mounting larvae ventral side up, in 30-mm glass-bottom petri dishes (MatTek, Ashland, MA) in 1.2% low-melt agarose (GeneMate). When agarose had set (∼10 min), mounted larvae were covered with egg water and placed in a 28 °C incubator immediately adjacent to the microscope for ∼30 min. Mounted larvae were then removed one at a time and imaged immediately on a Stemi SV11 dissecting microscope (Carl Zeiss, Thornwood, NY) outfitted with a CCD camera at 20 frames per second (Toshiba IK-C43H 19). Measurements were taken from the first 10 s of video. Videos were then analysed with Fiji software (www.fiji.sc/) and atrial and ventricular fractional shortening was calculated as described^34^. Ten fish were measured per group.

### Zebrafish heart electron microscopy

For electron microscopy, E17K transgenic and WT adult zebrafish hearts were dissected and immediately fixed in 2% glutaraldehyde, 1% paraformaldehyde in 0.1 M sodium cacodylate buffer pH 7.4, post-fixed in 2% osmium tetroxide in the same buffer, en bloc stained with 2% aqueous uranyl acetate, dehydrated in acetone, infiltrated and embedded in LX-112 resin (Ladd Research Industries, Burlington, VT). Specimens were ultrathin sectioned on a Reichert Ultracut S ultramicrotome and counter stained with 0.8% lead citrate. Grids were examined on a JEOL JEM-1230 transmission electron microscope (JEOL USA, Inc., Peabody, MA) and photographed with the Gatan Ultrascan 1000 digital camera (Gatan Inc., Warrendale, PA). Evaluation of images was performed by a cardiac pathologist (P. Ursell, UCSF) blinded to the labelling of samples, nature of the experimental perturbation and the clinical phenotype of affected individuals.

### Quantitative PCR

Total RNA was extracted from zebrafish tissues using Trizol reagent (GibcoBRL), purified using the Zymo RNA Clean & Concentrator-5 kit (Zymo Research), treated with DNAse (Qiagen) and reverse transcribed, as previously described^35^, using SuperScript IV reverse transcriptase (ThermoFisher). Real-time PCR was performed using an ABI Prism 7900HT Fast Real-Time PCR System (Applied Biosystems) and SensiFAST SYBR Hi-ROX Kit (Bioline). qPCR was performed comparing the expression of *cmlc1* in the E17K transgenic relative to the WT transgenic at 5 d.p.f. (3 technical replicates per biological replicate; 3 biological replicates, 40 larvae per biological replicate). qPCR was also performed comparing the select genes in atria dissected from adult WT transgenic and E17K transgenic fish (three technical replicates per biological replicate; two biological replicates with one atrium per biological replicate; the third biological replicate was a pool of three adult atria). In addition to *cmlc1*, a subset of other genes were selected based on their cardiac expression, documented cardiac phenotypes in zebrafish and/or reported association with human AF. Exon-spanning primers were chosen where possible; no-RT controls for each biological replicate further confirmed insignificant genomic DNA contamination. Fold expression was calculated using the delta–delta Ct method, using elongation factor I alpha (*eef1a1|1*) as a reference gene. Statistical significance for each gene was calculated using a two-tailed *t*-test.

### Molecular modelling

Secondary structure of WT and mutant N-termini of Myl4 was modelled using the QUARK protein structure prediction algorithm^23^. To model the interaction of the N-terminal region of MYL4 with F-actin, we adopted the approach described by Aydt *et al*.^36^ Briefly, the modelling utilizes known crystal structures demonstrating the 3D structure of actin (PDB 1ATN) and the crystal structure representing the interaction of chick myosin heavy chain, ELC and regulatory light chain with actin (PDB 2MYS). These crystal structures provide information on 116,559 atomic coordinates^36^. Aydt *et al*.^36^ modelled the binding of the N-terminal region of chick ELC (MLE1) onto these crystal structures. In our model, we substituted the homologous (76% identity) N-terminal region of *MYL4* for the N-terminal region of chick ELC (MLE1) studied by Aydt *et al*^36^. and used the SWISS-MODEL program and the same atomic coordinates to model the tertiary structure of the interaction of WT and mutant N-termini of MYL4 with F-actin^37^. Residues within actin and MYL4 with the potential to interact with E11 on MYL4 were determined by examining the area within 5 Å of E11. Residues with hydrophobic properties or side chains incapable of interacting with E11 were discarded as possibilities. This analysis resulted in a predicted electrostatic interaction of MYL4-E11 with K358 on actin. The effect on the Myl4-actin interaction caused by substituting the positively charged K11 for negatively charged E11 was also modelled in a similar fashion^38^.

## Additional information

**Accession codes:** The *MYL4* mutation encoding p.Glu11Lys has been deposited in the NCBI ClinVar database under accession # SCV000264340.

**How to cite this article:** Orr, N. *et al*. A mutation in the atrial-specific myosin light chain gene (*MYL4*) causes familial atrial fibrillation. *Nat. Commun.* 7:11303 doi: 10.1038/ncomms11303 (2016).

## Supplementary Material

Supplementary Information Supplementary Figures 1-2 and Supplementary Tables 1-2

## Figures and Tables

**Table 1:** Clinical characteristics of affected individuals.

Clinical characteristics							ECG				Echocardiogram		
ID	Sex	Age	Presentation	Age at AF diagnosis	AF status	Conduction disease	Sinus HR (b.p.m.)	Av. AF rate (b.p.m.)	PR (ms)	QRS (ms)	LVEF (%)	LAVI (ml m^−2^)	LAFI (%)
II:2	F	53	Palpitations	32	Permanent AF	CHB/PPM	70	40*	—	72	68	40	10
II:3	M	50	Asymptomatic	38	Permanent AF	Uncertain	—	80	—	84	68	38	21
III:1	F	25	Asymptomatic	22	Permanent AF	Uncertain	—	50	—	76	72	25	30
III:2	F	29	Palpitations	25	PAF	1° AVB	65†	55	216	88	67	38	35
III:3	F	32	Palpitations	26	PAF	1° AVB	74	59	336	88	66	33	39
III:5	F	20	Asymptomatic	—	NSR	1° AVB	80	—	200	80	72	24	52

AF, atrial fibrillation; Av.AF rate, average heart rate during AF (determined by ECG over 10 second interval while off medication); AVB, atrial–ventricular block; b.p.m., beats per minute; CHB, complete heart block; ECG, electrocardiography; F, female; HR, heart rate; ID, subject (index) ID; LAFI, left atrial function index (normal 54±17% (ref. 8)); LAVI, left atrial volume index (normal <28 ml m^−2^, severely enlarged >39 ml m^−2^); LVEF, left ventricular ejection fraction; M, male; NSR, normal sinus rhythm; PAF, paroxysmal AF; PPM, permanent pacemaker.

^*^Junctional escape rhythm.

^†^Profound sinus bradycardia and frequent pauses observed on 48-h ambulatory monitoring.

**Table 2:** ECG characteristics of WT transgenic and E17K transgenic adult zebrafish.

	WT transgenic	E17K transgenic	*P* value
RR interval	587±94 msec	780±122 msec	0.009
Average HR	105±17 b.p.m.	82±14 b.p.m.	0.004
HR variability	5.4%	21%	0.0007
P-wave duration	26±4 msec	34±6 msec	0.004
PR interval	74±6 msec	70±17 msec	0.4

b.p.m., beats per minute; ECG, electrocardiogram; HR, heart rate; WT, wild type.
