# Supplementary material for: A mutation in the atrial-specific myosin light chain gene (MYL4) causes familial atrial fibrillation
Source: Nat Commun. 2016 Apr 12;7:11303. doi: 10.1038/ncomms11303 (PMC4832069; doi:10.1038/ncomms11303)
Supplement: Supplementary Information — Supplementary Figures 1-2 and Supplementary Tables 1-2 [file ncomms11303-s1.pdf]

Supplemental Information

Supplementary Figure 1

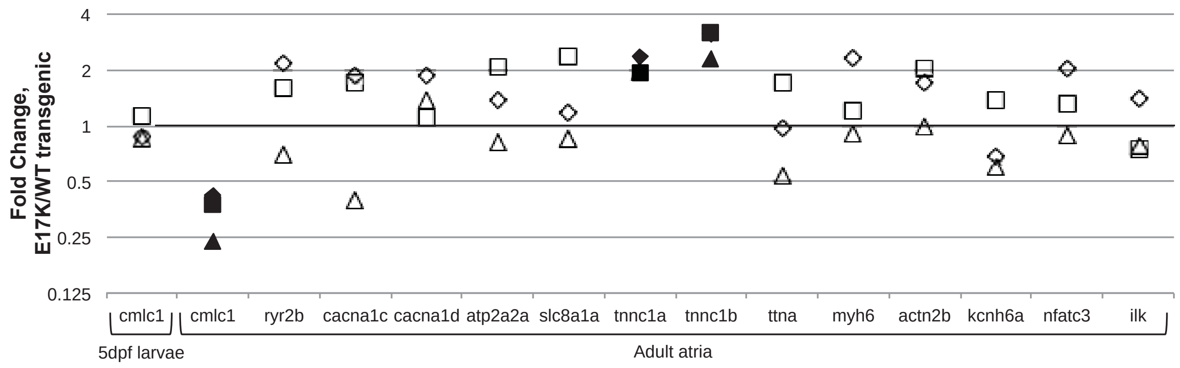

Relative expression of select genes in E17K transgenic compared to WT transgenic. *cmlc1* was assayed at 5dpf, when larvae exhibit myofibrillar disarray but no changes in chamber size or function, and also at adult stage from dissected atria. Other select genes were also assayed in adult atrium. Biological replicates are plotted for each gene; different replicates are designated by square, diamond, and triangle symbols. Black symbols represent statistically significant fold changes in the E17K transgenic compared to the WT transgenic (using a two-tailed t-test, *p* values for adult *cmlc1*, *tnnc1a*, and *tnnc1b* are p=0.003, p=0.003, p=0.001, respectively). *cmlc1*, cardiac myosin light chain 1; *ryr2b*, ryanodine receptor 2b; *cacna1c*, L-type voltage-dependent calcium channel alpha subunit 1c; *cacna1d*, L-type voltage-dependent calcium channel alpha subunit 1d; *atp2a2a*, calcium-transporting cardiac ATPase; *slc8a1a*, sodium-calcium exchanger member 1a; *tnnc1a*, troponin C type 1a; *tnnc1b*, troponin C type 1b; *ttna*, titin a; *myh6*, cardiac myosin heavy chain 6 alpha; *actn2b*, alpha actinin; *kcnh6*, potassium voltage-gated channel 6a; *nfatc3c*, calcineurin-dependent nuclear factor of activated T cells 3a; *ilk*, integrin-linked kinase.

Supplementary Figure 2

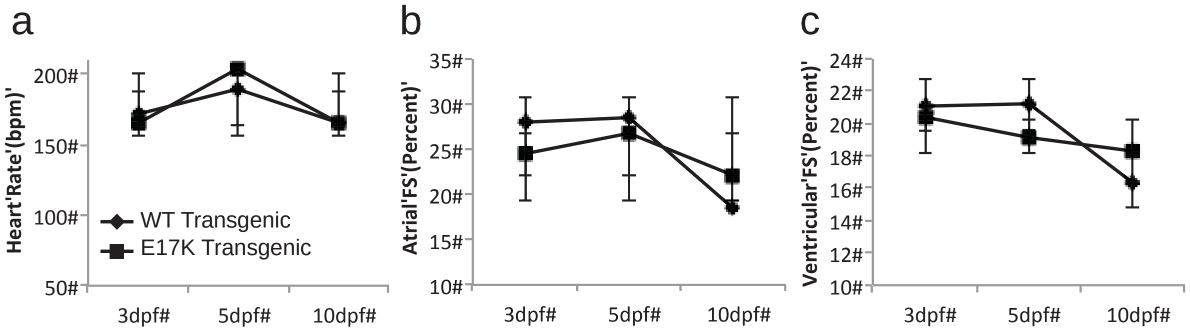

Function of larval E17K transgenics is preserved. (a-c) Heart rate, atrial fractional shortening, and ventricular fractional shortening of mutant transgenic zebrafish larvae were similar to WT transgenic larvae at 3, 5, and 10 dpf.

Supplementary Table 1. Novel protein-altering variants shared by affected individuals.

Gene	Identified AA change	Chr	Position (hg19)	Base change	Conservation score*	Polyphen-2 score	Mutation Taster[†]
MYL4	E11K	17	45286819	G>A	High (0.999)	Possibly Damaging (0.608)	Disease causing (0.997)
IL13RA2	F357L	X	114239807	A>G	Low (0.0)	Benign (0)	Polymorphism (1)
SCN7A	S812G	2	167288986	T>C	Moderate (0.315)	Possibly Damaging (0.904)	Polymorphism (1)
FNBP1	T576I	9	132658236	G>A	Moderate (0.343)	Benign (0)	Polymorphism (0.743)
A2ML1	V1394A	12	9021759	T>C	High (1.0)	Possibly Damaging (0.604)	Polymorphism (0.996)
KRT39	S335F	17	39116746	G>A	High (1.0)	Probably Damaging (1)	Disease Causing (1)

*Maximum score = 1 (based on phastCons analysis)
[†]Value given is the probability of the prediction's accuracy

Supplementary Table 2. Gene Ontology of novel protein altering variants.

Gene	Protein	Highest Tissue Expression	Cellular Component	Molecular Function	Biological Process
MYL4	essential atrial myosin light chain 1	atrial myocardium	intracellular, actin cytoskeleton	structural activity	muscle contraction
IL13RA2	Interleukin 13 receptor, alpha 2	testis	cell membrane	cytokine receptor activity	immune system response
SCN7A	Type VIIA voltage-gated sodium channel	brain	cell membrane	cation transmembrane transporter	Central Nervous System, sodium concentration sensor
FNBP1	formin binding protein 1	thymus, white blood cells	intracellular, lysozomes	lipid binding	clathrin-mediated endocytosis
A2ML1	alpha-2-macroglobulin-like-1	epithelium, esophagus	extracellular space	peptidase inhibitor activity	regulation of endopeptidase activity
KRT39	type 1 hair keratin (keratin-39)	epithelium	intermediate filaments	structural activity	cellular structure
